# Ivabradine protects rats against myocardial infarction through reinforcing autophagy via inhibiting PI3K/AKT/mTOR/p70S6K pathway

**DOI:** 10.1080/21655979.2021.1925008

**Published:** 2021-05-11

**Authors:** Yingnan Dai, Yeping Chen, Guoqian Wei, Li Zha, Xueqi Li

**Affiliations:** Department of Cardiology, The Fourth Affiliated Hospital of Harbin Medical University, Harbin, P.R. China

**Keywords:** Ivabradine, myocardial infarction, autophagy, PI3K/AKT/MTOR/p70S6K

## Abstract

Ivabradine (Iva), a heart rate reducing agent that specifically inhibits the pacemaker *I(f)* ionic current, has been demonstrated to be cardioprotective in many cardiovascular diseases. Autophagy is an evolutionarily conserved metabolic process that regulates cardiac homeostasis. This study is aimed to explore whether autophagy is functionally involved in the cardioprotective effect of Iva in a rat model of myocardial infarction (MI). We observed that Iva treatment (po, 10 mg/kg/day) showed significant recovery on the hemodynamics parameters in MI rats, including left ventricular systolic pressure, left ventricular end diastolic pressure, and maximal ascending/descending rate of left ventricular pressure. Also, Iva treatment dramatically decreased infarct size, inhibited myocardial apoptosis, and reduced the levels of pro-inflammatory cytokines tumor necrosis factor (TNF)-α, interleukin (IL)-1β and IL-6 in MI rats. Moreover, Iva treatment enhanced autophagy and inhibited PI3K/AKT/mTOR/p70S6K pathway in MI rats. Simultaneously, we observed that autophagy enhancer rapamycin (ip, 10 mg/kg/day) showed similar cardioprotective effects with Iva. Furthermore, we observed that addition of autophagy inhibitor 3-methyladenine (ip, 10 mg/kg/day) counteracted the therapeutic effect of Iva, addressing that Iva attenuated post-MI cardiac injury by enhancing autophagy. In summary, these findings demonstrated that Iva attenuated MI in rats by enhancing autophagy, and PI3K/AKT/mTOR/p70S6K pathway might be involved in the process. Autophagy activation by Iva may be a potential therapeutic strategy for the treatment of MI.

## Graphical Abstract

**Figure:**
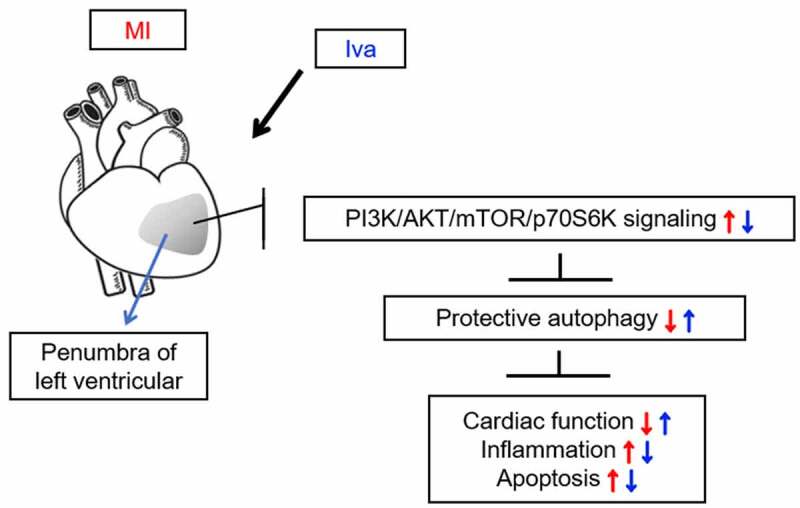

## 1 Introduction

Myocardial infarction (MI) is a severe cardiovascular disease caused by temporary or permanent coronary arterial occlusion, leading to negative myocardial remodeling, contractile dysfunction, myocardial necrosis, arrhythmias, and even heart failure [1,2]. Despite rapid advancements have been made, MI remains a leading cause of morbidity and mortality worldwide, and the increased prevalence of MI is placing a heavy burden to the society [3]. Generally, patients who survive from acute MI usually face an increased risk of post-MI left ventricular (LV) remodeling and heart failure [4]. The adverse LV remodeling has been shown to be closely associated with the poor outcome of MI [5]. Hence, it is of great significance to develop effective alternative therapies against LV dysfunction in the treatment of MI.

During the past decades, pharmacological and interventional management against myocardial injury have been dramatically improved [6,7]. Notably, Ivabradine (Iva), a highly selective blocker of specific ion channels that conduct the hyperpolarization-activated I(f) current, has attracted our attention [8]. Iva is a novel and currently the only hyperpolarization-activated cyclic nucleotide-gated channel 4 (HCN4) inhibitor used clinically for chronic stable angina and chronic heart failure [9]. It has been revealed that Iva slows heart rate by reducing the slope of the diastolic depolarization of pacemaker action potential, without affecting cardiac inotropy or systemic vascular resistance [10]. Emerging evidence has proven the efficacy and safety of Iva in other cardiovascular diseases such as inappropriate sinus tachycardia [11] and ventricular arrhythmias [12]. However, the guidelines of Iva in the application of acute coronary syndromes has not been established. Based on the data from some preclinical or clinical studies, it is known that early use of Iva combined with standard therapy such as β-blocker attenuate the LV remodeling after primary percutaneous coronary intervention in patients with acute ST‐segment elevation MI [13]. Also, Iva promotes angiogenesis and reduces cardiac hypertrophy in a mouse model with MI [14]. However, the effect and mechanism of Iva in MI still warrant further investigation.

Autophagy is an evolutionarily conserved metabolic process that mediates the degradation of superfluous, senescent or damaged cellular components [15,16]. A growing body of evidence demonstrates that autophagy is vital for cardiac homeostasis and function [17]. In most conditions, autophagy preserves cardiac function and reduces myocardial injury through degrading misfolded proteins, alleviating mitochondrial dysfunction and inhibiting oxidative stress [18,19]. These findings implicate that the enhancement of autophagy function is beneficial for MI treatment [20].

In this work, we investigate the therapeutic effectiveness and mechanism of Iva on MI in a rat model. Our novel findings demonstrate that Iva protects rats against MI by specifically enhancing autophagy, which might be associated with the inhibition of PI3K/AKT/mTOR/p70S6K pathway.

## 2 Materials and methods

### 2.1 Ethics statement

All animal use procedures were approved by the Ethical Committee of the Fourth Affiliated Hospital of Harbin Medical University and performed following the Guide for the Care and Use of Laboratory Animal (Eighth Edition). All surgery was performed under sodium pentobarbital anesthesia, and all efforts were made to minimize suffering.

### 2.2 Establishment of rat MI model

One hundred and twenty eight-week-old male Sprague-Dawley rats (Changsheng Biotechnology Co., Ltd., Liaoning, China) were kept in standard cages under controlled temperature and humidity with free access to food and water.

After fed adaptably for a week, 96 rats were anesthetized using sodium pentobarbital (50 mg/kg, i.p). A small-animal ventilator (HX-101E, Chengdu Taimeng Software Co., Ltd., Chengdu, China) was applied to maintain rat respiration. After the rat’s breathing was stable, an incision was made on the rat’s left chest along the second and third rib to expose the heart, and a surgical ligation on the left anterior descending coronary artery was performed. The surgical incision was carefully sutured, the ventilator was removed, and the rats were placed back to the terrarium. These rats were randomly divided into four groups (N = 24 per group): MI group, MI + Iva group, MI + Iva + 3-methyladenine (3-MA) group and MI + rapamycin (Rap) group. At the same time, the rest 24 normal rats were assigned as the Sham group. Rats in the Sham group were only exposed to the heart without ligation. Second day after surgery, rats in the MI + Iva group and MI + Iva + 3-MA group received an intragastric administration of 10 mg/kg Iva (Aladdin Biochemical Technology Co.,Ltd., Shanghai, China) daily for seven consecutive days. Rats in MI + rapamycin group received an intraperitoneal injection of 2 mg/kg Rap (Aladdin Biochemical Technology Co.,Ltd.) daily for 7 days, and rats in MI + Iva +3-MA group received an additional intraperitoneal injection of 15 mg/kg 3-MA (Aladdin Biochemical Technology Co.,Ltd.) daily for 7 days. Rats in the Sham group received equal amount of solvent. The procedure of animal experiment was shown in a flow chart (supplementary Fig. 1).

Seven days after MI surgery, six of rats from each group were anesthetized using 1% sodium pentobarbital (50 mg/kg, i.p) and hemodynamics parameters were subsequently examined. Then these rats were sacrificed using sodium pentobarbital (200 mg/kg, i.p) and the hearts were isolated and stored at −70°C for Western blot analysis. The rest of rats in each group were sacrificed using sodium pentobarbital and the hearts were carefully isolated. Six of hearts in each group were subjected to 2,3,5-triphenyltetrazolium (TTC) staining. Another six of hearts in each group were fixed with 4% paraformaldehyde and subjected to hematoxylin and eosin (HE) staining and terminal deoxynucleoitidyl transferase-mediated dUTP-biotin nick end labeling (TUNEL) staining. The rest six hearts in each group were stored at −70°C for enzyme-linked immunosorbent assay (ELISA) and quantitative real-time polymerase chain reaction (qRT-PCR) analysis.

### 2.3 Detection of hemodynamic parameters

Twenty-four hours after the last dose of Iva or rapamycin, rats were anesthetized with 1% sodium pentobarbital and then placed on the operating table. A left ventricular intubation was performed via the right common carotid artery for blood pressure monitoring. The left ventricular systolic pressure (LVSP), left ventricular end diastolic pressure (LVEDP) and maximal ascending/descending rate of left ventricular pressure over time (± dp/dt_max_) were measured using a biological and functional experimental system (BL-420, Chengdu Taimeng Software).

### 2.4 Detection of infarction size

The isolated hearts were cut into 2-mm-thick sections and immediately immersed in TTC solution (Solarbio Science & Technology Co., Ltd, Beijing, China) at 37°C for 30 min. The staining results were photographed under a digital camera. Pale color represents the infarction tissue. Infarction area (%) was determined by white infarct area/whole slice area using Image-Pro Plus 6.0 (Media Cybernetics, Inc., USA).

### 2.5 Detection of pathological changes

The pathological changes in heart tissues were examined using HE staining according to manufacturer’s protocols. The staining results were observed under an optical microscope (BX53, Olympus Corporation, Tokyo, Japan) at 200 × magnifications.

### 2.6 Detection of apoptotic cardiomyocytes

Apoptotic cardiomyocytes was detected using a TUNEL staining apoptotic kit (Roche Group, Basel, Switzerland) following the manufacturer’s protocols. The results were observed under an optical microscope (BX53, Olympus Corporation, Tokyo, Japan) at 400 × magnifications.

### 2.7 ELISA

Levels of pro-inflammatory factors tumor necrosis factor (TNF)-α, interleukin (IL)-1β and IL-6 were detected using commercial ELISA kits (Boster Biological Technology Co., Ltd, Hubei, China) according to the manufacturer’s protocols.

### 2.8 qRT-PCR

Total RNA was isolated using TRIpure reagent (Bioteke Corporation, Beijing, China) and quantified using a UV spectrophotometer (NanoDrop 2000, Thermo Fisher Scientific, Waltham, USA). Complementary DNA (cDNA) was synthesized using Super M-MLV reverse transcriptase (Bioteke Corporation). qRT-PCR was performed using 2× Power Taq PCR MasterMix (Bioteke Corporation) and SYBR Green (Sigma-Aldrich Corp., Missouri, USA) on a fluorescence quantitative PCR instrument. β-actin was used as an internal control. The primers were: TNF-α (forward), CGGAAAGCATGATCCGAGAT; TNF-α (reverse), AGACAGAAGAGCGTGGTGGC. IL-1β (forward), TTCAAATCTCACAGCAGCAT; IL-1β (reverse), CACGGGCAAGACATAGGTAG. IL-6 (forward), AACTCCATCTGCCCTTCA; IL-6 (reverse), CTGTTGTGGGTGGTATCCTC. β-actin (forward), GGAGATTACTGCCCTGGCTCCTAGC; β-actin (reverse), GGCCGGACTCATCGTACTCCTGCTT.

### 2.9 Western blot analysis

Total protein was isolated using Western & IP lysate (Beyotime Biotechnology, Shanghai, China) buffer. BCA protein assay kit (Beyotime Biotechnology) was applied to determine protein concentration. Equal amount of protein was separated using sodium dodecyl sulfate-polyacrylamide gel electrophoresis and then blotted to the polyvinylidene fluoride membranes (Millipore corp., Massachusetts, USA). Membranes were then blocked with 5% skimmed milk (Yili Industrial Group Co., Ltd., Inner Mongolia, China) and incubated with primary antibodies against p62 (1:1000, cat. No. 39,749, Cell Signaling Technology, Inc., Massachusetts, USA), LC3 (1:1000, cat. No. 4108, Cell Signaling Technology), Beclin-1 (1:1000, cat. No. 3495, Cell Signaling Technology), ATG-5 (1:2000, cat. No. 12,994, Cell Signaling Technology), ATG-7 (1:500, cat. No. 8558, Cell Signaling Technology), p-AKT (1:500, cat. No. 4060, Cell Signaling Technology), AKT (1:1000, cat. No. 4691, Cell Signaling Technology), p-PI3K (1:1000, cat. No. AF3242, Affinity Biosciences, Cincinnati, OH, USA), PI3K (1:1000, cat. No. 4257, Cell Signaling Technology), p-mTOR (1:500, cat. No. 5536, Cell Signaling Technology), mTOR (1:1000, cat. No. 2983, Cell Signaling Technology), p-p70S6K (1:1000, cat. No. 97,596, Cell Signaling Technology), p70S6K (1:2000, cat. No. 9202, Cell Signaling Technology) or β-actin (1:1000, cat. No. sc-47,778, Santa Cruz Biotechnology, Inc., California, USA) at 4°C overnight. After washed with TBST for three times, membranes were then incubated with secondary antibodies against goat-anti-rabbit IgG (1:5000, cat. No. A0208, Beyotime) or goat-anti-mouse IgG (1:5000, cat. No. A0216, Beyotime) for 45 min at 37°C. The blots were visualized by enhanced chemiluminescence. Quantitation of blots were ensured using Image-Pro Plus software and the results were normalized to the densitometry of β-actin.

### 2.10 Immunohistochemistry

The fixed myocardial tissues were embedded in paraffin and cut into 5-μm-thick sections. Sections were deparaffinized, hydrated and then subjected to antigen retrieval. Then the sections were incubated with primary antibody against ATG-5 (1:100, cat. No. A11427, ABclonal Technology, Hubei, China) in a wet box at 4°C overnight, followed by incubation with secondary antibody against goat anti-rabbit IgG (1:500, Thermo Fisher Scientific) for 30 min at 37°C. DAB solution (Solarbio) was used for color rendering and the results were observed under an optical microscope at 400 × magnifications.

### 2.11 Statistical analysis

GraphPad Prism version 8.0.2 was used to calculate the significance among groups. Data were presented as mean ± SD. Normality of the distribution within the groups was evaluated using the Kolmogorov-Smirnov test. All data satisfied the assumptions of normality and homogeneity of variance. Data were analyzed using one-way analysis of variance followed by Tukey’s HSD post-hoc test. A p-value <0.05 was considered as statistically significant difference.

## 3 Results

### 3.1 Effects of Iva on the cardiac hemodynamics of MI rats

**Figure 1.**
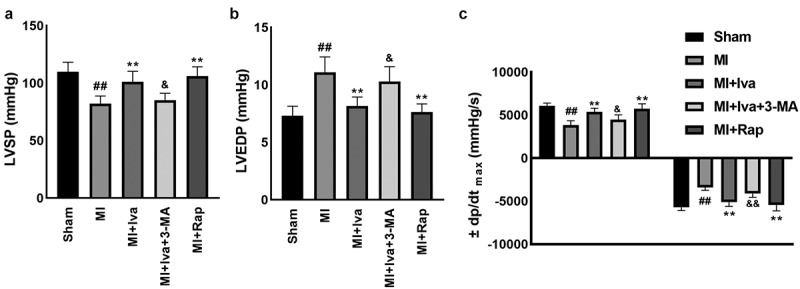
Effect of ivabradine on LVSP, LVEDP, and ± dp/dt_max_ of MI rats. (a) Left ventricular systolic pressure (LVSP). (b) Left ventricular end diastolic pressure (LVEDP). (c) maximal ascending/descending rate of left ventricular pressure over time (± dp/dt_max_). n = 6 rats/group. Data were expressed as mean ± SD; ^##^*P* < 0.01 vs Sham group; **P < 0.01 vs MI group; ^&^*P* < 0.05, ^&&^*P* < 0.01 vs MI+Iva group. Abbreviations: 3-MA, 3-methyladenine; Iva, ivabradine; MI, myocardial infarction; Rap, rapamycin

In this work, we established a MI model *in vivo* by performing a ligation surgery in the left anterior descending coronary artery in rats. Iva, Rap or 3-MA were administered alone or in combination as to assess their effects on MI. To assess the therapeutic effect of Iva on cardiac dysfunction post MI surgery, we detected the hemodynamic parameters including LVSP, LVEDP, and ±dp/dt_max_. As shown in Figure 1, the levels of LVSP and ±dp/dt_max_ were significantly reduced, whereas the level of LVEDP was significantly elevated in rats of MI group compared with the Sham group, indicating that the cardiac function was notably injured post MI surgery. Moreover, Iva treatment significantly elevated the LVSP and ±dp/dt_max_ levels and decreased the LVEDP level, addressing that Iva played a protective role against MI-associated cardiac dysfunction. Besides, to assess the role of autophagy on cardiac function during MI, we observed that the LVSP and ±dp/dt_max_ levels were significantly increased, while LVEDP level was significantly decreased in rats of the MI + Rap group compared to MI group, indicating autophagy enhancement had a protective role against MI. Moreover, we observed that the LVSP and ±dp/dt_max_ levels were markedly decreased, while LVEDP level was increased in rats of the MI + Iva + 3-MA group compared to MI + Iva group, suggesting that autophagy inhibition abolished the cardioprotective effect of Iva on MI. These results indicated that Iva improved cardiac function by enhancing autophagy.

### 3.2 Effect of Iva on the cardiac infarct size of MI rats

**Figure 2.**
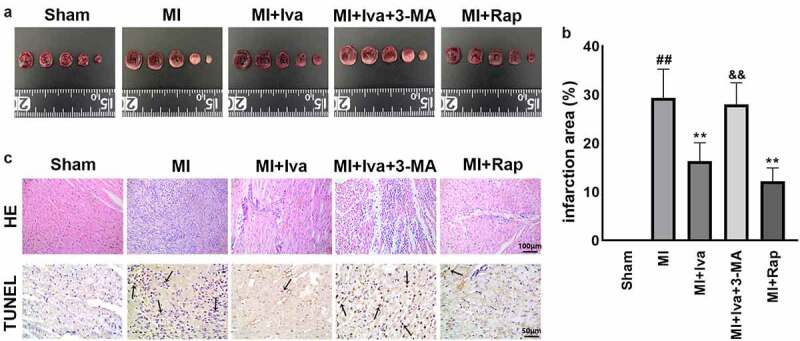
Effect of ivabradine on infarction size and cardiomyocyte apoptosis of MI rats. (a) Representative images of 2,3,5-triphenyltetrazolium chloride (TTC) staining. (b) Quantitation of infarction size. (c) Representative images of terminal deoxynucleoitidyl transferase-mediated dUTP-biotin nick end labeling (TUNEL) assay. Black arrows indicated TUNEL-positive cardiomyocytes. n = 6 hearts/group. Data were expressed as mean ± SD; ^##^*P* < 0.01 vs Sham group; **P < 0.01 vs MI group; ^&&^*P* < 0.01 vs MI+Iva group. Abbreviations: 3-MA, 3-methyladenine; Iva, ivabradine; MI, myocardial infarction; Rap, rapamycin

To further evaluate the therapeutic effects of Iva and the role of autophagy in MI, the cardiac infarct size of rats in each group were detected using TTC staining. As shown in Figure 2a and 2b, the infarction area was dramatically increased in MI group compared to Sham group, whereas the infarction area was significantly decreased in both MI + Iva group and MI + Rap group compared to MI group. Moreover, the infarction area was increased again after addition of 3-MA. These results indicated that Iva reduced the myocardial infarct size by enhancing autophagy.

### 3.3 Effect of Iva on the cardiac pathological changes of MI rats

Besides, the pathological changes in cardiac tissues were also detected by HE staining and TUNEL staining (Figure 2c). We observed that 7 days post MI surgery, the soma of infarcted myocardial cells showed deformation, and increased apoptotic cells were observed in MI group compared to Sham group. Simultaneously, both Iva treatment and Rap treatment effectively alleviated myocardial necrosis and apoptosis, whereas 3-MA addition counteracted the protective effect of Iva against myocardial necrosis and apoptosis. These results suggested that Iva attenuated the MI-induced pathological changes in rats by enhancing autophagy.

### 3.4 Effect of Iva on the production of inflammatory cytokines of MI rats

**Figure 3.**
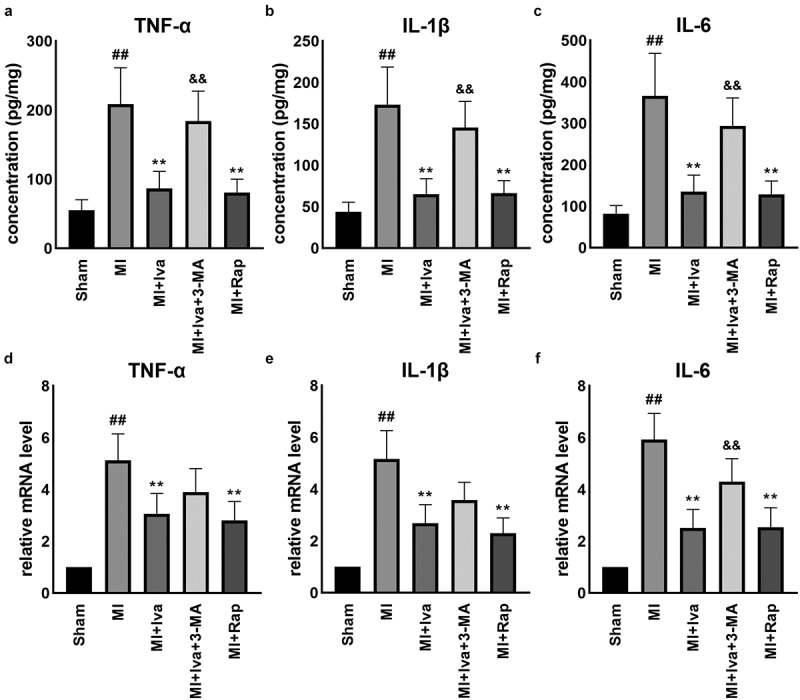
Effect of ivabradine on TNF-α, IL-1β, and IL-6 production of MI rats. Serum concentration of TNF-α (a), IL-1β (b), and IL-6 (c). mRNA expression levels of TNF-α (d), IL-1β (e), and IL-6 (f) in myocardial tissues. n = 6 hearts/group. Data were expressed as mean ± SD; ^##^*P* < 0.01 vs Sham group; **P < 0.01 vs MI group; ^&&^*P* < 0.01 vs MI+Iva group. Abbreviations: 3-MA, 3-methyladenine; Iva, ivabradine; MI, myocardial infarction; Rap, rapamycin

As well-known, cardiac inflammation positively links with MI. Therefore, we further detected cardiac inflammation in this study. The results of ELISA and qRT-PCR assays showed that the expression of pro-inflammatory cytokines TNF-α, IL-1β and IL-6 was significantly increased in cardiac tissues post MI operation. Both Iva treatment and Rap treatment significantly reduced the expression of these cytokines, while 3-MA addition partially abolished the effect of Iva (Figure 3). These data indicated that Iva suppressed inflammatory response post MI by enhancing autophagy.

### 3.5 Effect of Iva on the autophagy function of MI rats

**Figure 4.**
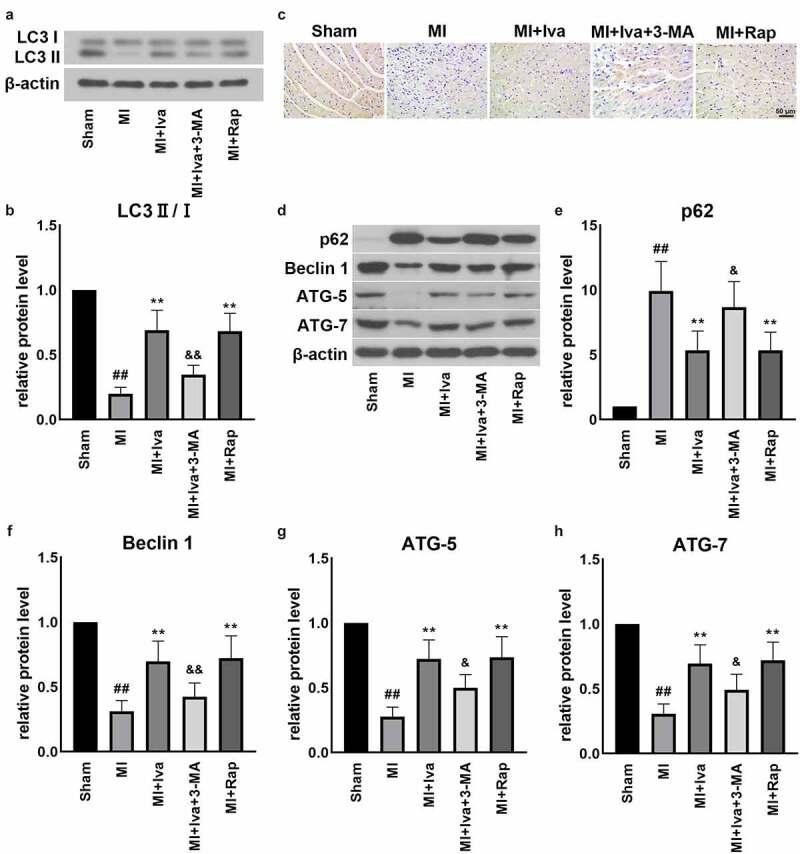
Effect of ivabradine on autophagy function of MI rats. (a) Representative Western blot (WB) bands of LC3II/I. (b) Quantification of LC3II, as normalized to LC3I. (c) Representative immunohistochemistry (IHC) staining images of ATG-5. (d) Representative WB bands of p62, Beclin1, ATG-5 and ATG-7. Quantification of p62 (e), Beclin1 (f), ATG-5 (g) and ATG-7 (h), as normalized to β-actin. n = 6 hearts/group. Data were expressed as mean ± SD; ^##^*P* < 0.01 vs Sham group; **P < 0.01 vs MI group; ^&^*P* < 0.05, ^&&^*P* < 0.01 vs MI+Iva group. Abbreviations: 3-MA, 3-methyladenine; Iva, ivabradine; MI, myocardial infarction; Rap, rapamycin

To further verify the effect of Iva on autophagy, we detected the protein expression of autophagy-related factors, including LC3, ATG-5, p62, Beclin 1 and ATG-7 (Figure 4). We observed that in the MI group, the expression of LC3 ∥, ATG-5, Beclin 1 and ATG-7 was dramatically down-regulated, whereas the expression of p62 was markedly up-regulated, compared to Shan group. Reversely, both Iva treatment and Rap treatment significantly elevated the LC3 II, ATG-5, Beclin 1 and ATG-7 levels and reduced the p62 level. Moreover, addition of 3-MA blocked the effect of Iva on the expressions of these proteins, implicating that Iva enhanced the injured autophagy function post MI.

### 3.6 Effect of Iva on the PI3K/AKT/mTOR/p70S6K pathway of MI rats

**Figure 5.**
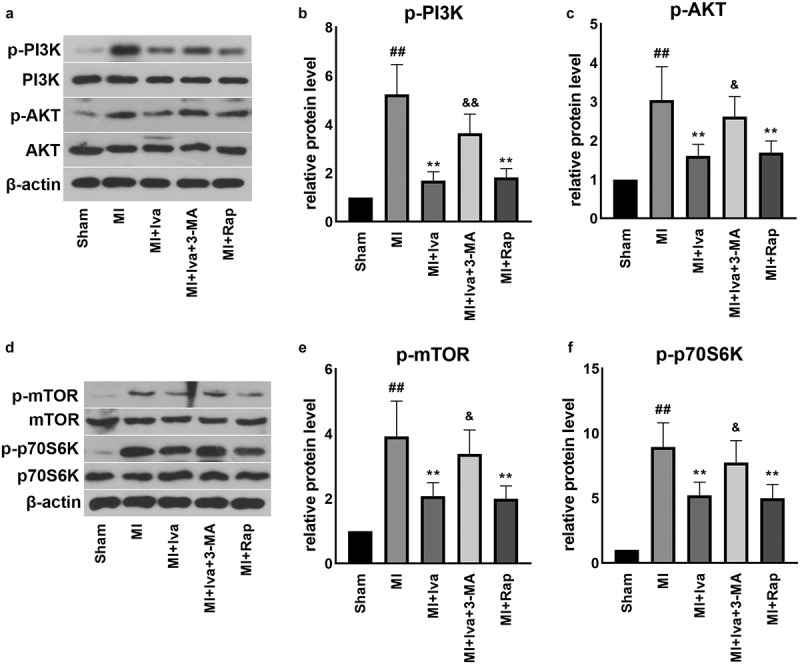
Effect of ivabradine on AKT/mTOR/p70S6K pathway of MI rats. (a) Representative WB bands of p-PI3K, PI3K, AKT and p-AKT. Quantification of p-PI3K (b) and PI3K (c), as normalized to β-actin. (d) Representative WB bands of mTOR, p-mTOR, p70S6K and p-p70S6K. Quantification of p-PI3K (e) and PI3K (f), as normalized to β-actin. n = 6 hearts/group. Data were expressed as mean ± SD; ^##^*P* < 0.01 vs Sham group; **P < 0.01 vs MI group; ^&^*P* < 0.05 vs MI+Iva group. Abbreviations: 3-MA, 3-methyladenine; Iva, ivabradine; MI, myocardial infarction; Rap, rapamycin

As autophagy is closely associated with PI3K/AKT/mTOR/p70S6K signaling, we further detected key proteins in this pathway (Figure 5). We found that the protein levels of p-PI3K, p-AKT, p-mTOR and p-p70S6K were all significantly increased in the cardiac tissues of MI group compared to those of the Sham group. On the contrary, both Iva treatment and Rap treatment effectively inhibited the expression of these proteins, whereas 3-MA addition successfully abolished the effect of Iva. These findings showed that Iva inhibited PI3K/AKT/mTOR/p70S6K signaling to enhance antophagy.

## 4 Discussion

As a unique pharmacological agent that specifically reduces heart rate without affecting cardiac inotropy, blood pressure, or cardiac conduction system function, Iva has been widely applied in the treatment strategies of multiple cardiovascular diseases clinically [8,9,21]. The current guidelines on the clinical use of Iva is mainly on the patients with stable angina pectoris whose symptoms are inadequately controlled by β-blockers, or patients with chronic heart failure and reduced ejection fraction. However, the clinical application of Iva in acute coronary syndromes has not been established [22]. Intriguingly, recent preclinical and clinical evidence has shown the protective effect of Iva against MI [14,23,24]. The involved mechanisms include cardiac renin-angiotensin-aldosterone system dysfunction [25], sarcoplasmic reticulum Ca^2+^-ATPase release [26], angiogenesis [14] and hyperpolarization-activated cyclic nucleotide-gated channel inactivation [27]. However, whether autophagy is functionally involved in the cardiac protection effect of Iva remains unknown. In this study, we assessed the effect of Iva in a rat cardiac injury model post MI, and explored whether autophagy is functionally involved in the process. We newly demonstrated that Iva exhibited its cardiac protection effect by activating autophagy post MI.

Autophagy is a critical cellular mechanism for cardiac cells to maintain homeostasis [17,28,29]. The application of enhancing autophagy function for treating cardiovascular diseases has attracted much attention of researchers recently. To investigate whether autophagy is involved in the cardiac protective role of Iva, we treated MI model rats with Rap or 3-MA [30,31] to enhance or blunt aautophagy, respectively We observed that both Iva and autophagy enhancer Rap exhibited significant cardiac protection effect against MI, as reflected by the significant recovery of hemodynamic parameters, alleviation of myocardial necrosis and apoptosis, and inhibition of cardiac inflammation. In contrast, addition of autophagy inhibitor 3-MA blocked the therapeutic effects of Iva on these aspects, further demonstrating that Iva attenuated the MI-induced cardiac injury by improving autophagy.

To further investigate the correlation between Iva and autophagy, autophagy-related proteins were detected in this work. Specifically, microtubule-associated protein 1A and 1B light-chain 3 (LC3) is a vital regulator in monitoring autophagy progression. Usually, during autophagy, the cytosolic LC3 (LC3-I) will be activated by ubiquitin-like ligases. The activated LC3-I will conjugate phospholipids to form LC3-phospholipid conjugate (LC3-II), which is a well-known promising marker for indicating autophagic activity [32]. In addition, numerous of studies illustrated that LC3 lipidation and truncation are intensely associated with the function of ATG proteins such as ATG-5 and ATG-7 [33–35], of which the latter acts as an important E1-like enzyme to modulate the aggregation of LC3-II. Besides, Beclin 1 is reported to promote lipid membrane extension, cargo recruitment, and autophagosome maturation, and serves as a critical autophagy regulator in heart [36]. Also, p62 is a critical autophagy adaptor protein to facilitate the selective degradation of protein cargo. The accumulation of p62 is considered as an indicator of defective autophagy [37]. In this study, we found that the levels of LC3-II, ATG-5, ATG-7 and Beclin 1 were reduced, whereas p62 level was increased post MI. These results indicated that autophagy funtion was dramatically injured post MI. Reversely, both Iva treatment and Rap treatment successfully restored the MI-induced disorders of these autophagy-related proteins. Furthermore, autophagy inhibitor 3-MA also abolished the activation effect of Iva on autophagy. All these findings implied that Iva activated autophagy to exert its cardiac protection effect.

Intriguingly, the enhanced cardiomyocyte autophagy is reported to be mediated by the PI3K/AKT/mTOR signaling pathway [38]. Previous work has reported that PI3K/AKT pathway activation is pathogenic in cardiac dysfunction and hypertrophy [39]. Moreover, a study reported that Iva prevented cardiac hypertrophy and fibrosis via inhibiting PI3K/AKT/mTOR pathway in an established transverse aortic constriction mouse model [40]. These findings suggest that the PI3K/AKT pathway-mediated autophagy inhibition is harmful to cardiac homeostasis. Consistently in this work, we demonstrated that the PI3K/AKT/mTOR/p70S6K pathway was activated during MI, whereas Iva effectively inhibited PI3K/AKT/mTOR/p70S6K signaling. Nevertheless, the role of AKT/mTOR signaling in MI progression is quite the opposite according to some other studies. For example, Meng et al. reported that downregulation of Annexin A3 promotes myocardial cell repair through activating PI3K/AKT signaling pathway [41]. Also, Wei et al. demonstrated that autophagy participates in the protection of 1,25-dihydroxyvitamin D3 in MI via activating PI3K/AKT/mTOR pathway [42]. These studies implicated that the activation of PI3K/AKT pathway triggers a protective mechanism against MI. Despite some controversial findings exist, the role of AKT/mTOR pathway in MI remains debatable. Possible explanations may be addressed on the excessive or injured autophagy happens during disease progression. According to our results, we observed that autophagy is negatively associated with PI3K/AKT/mTOR/p70S6K signaling in a rat MI model, which is consistent with some studies focused on the cardioprotective role of autophagy [43–45]. Therefore, we declared that Iva activated the injured autophagy via inhibiting PI3K/AKT/mTOR/p70S6K signaling in this study.

## Conclusion

In summary, our results provide evidence that Iva effectively protected rats from post-MI cardiac injury via enhancing autophagy function. A potential mechanism involved may be that Iva inhibited the transduction of PI3K/AKT/mTOR/p70S6K signaling pathway. This study may provide preclinical evidence for the application of Iva in treating MI.

## Supplementary Material

Supplemental Material

## Highlights

1. Ivabradine (Iva) has a cardioprotective role against myocardial infarction (MI).
2. Autophagy enhancement has a cardioprotective role against MI.
3. Iva enhanced the injured autophagy during MI in rats.
4. Autophagy inhibition abolished the cardioprotective role of Iva against MI.
