# Supplementary material for: Ivabradine protects rats against myocardial infarction through reinforcing autophagy via inhibiting PI3K/AKT/mTOR/p70S6K pathway
Source: Bioengineered. 2021 May 11;12(1):1826–37. doi: 10.1080/21655979.2021.1925008 (PMC8806854; doi:10.1080/21655979.2021.1925008)
Supplement: Supplemental Material [file KBIE_A_1925008_SM3779.zip › Document.rtf]

Supplementary Figure 1. Flow chart of animal experiments. Eight-week-old male Sprague-Dawley rats were subjected to left anterior descending artery ligation surgery to establish MI rat model. These MI rats were randomly divided into four groups: MI group, MI + Iva group, MI + Iva + 3-MA group, MI + Rap group. The rest of rats were assigned as the Sham group. Rats in Sham group received same surgery without ligation. On the second day after surgery, rats in MI + Iva group and MI + Iva + 3-MA groups received intragastric administration of 10 mg/kg Iva daily for 7 days. At the same time, rats in MI + rapamycin group received intraperitoneal injection of 2mg/kg Rap daily, and rats in MI + Iva +3-MA group received additional intraperitoneal injection of 15 mg/kg 3-MA daily. Rats in Sham group received equal amount of normal saline. N = 24 rats/group. Abbreviations: 3-MA, 3-methyladenine; Iva, ivabradine; MI, myocardial infarction; Rap, rapamycin.
