# Supplementary figures and images for: Ivabradine protects rats against myocardial infarction through reinforcing autophagy via inhibiting PI3K/AKT/mTOR/p70S6K pathway
Source: Bioengineered. 2021 May 11;12(1):1826–37. doi: 10.1080/21655979.2021.1925008 (PMC8806854; doi:10.1080/21655979.2021.1925008)

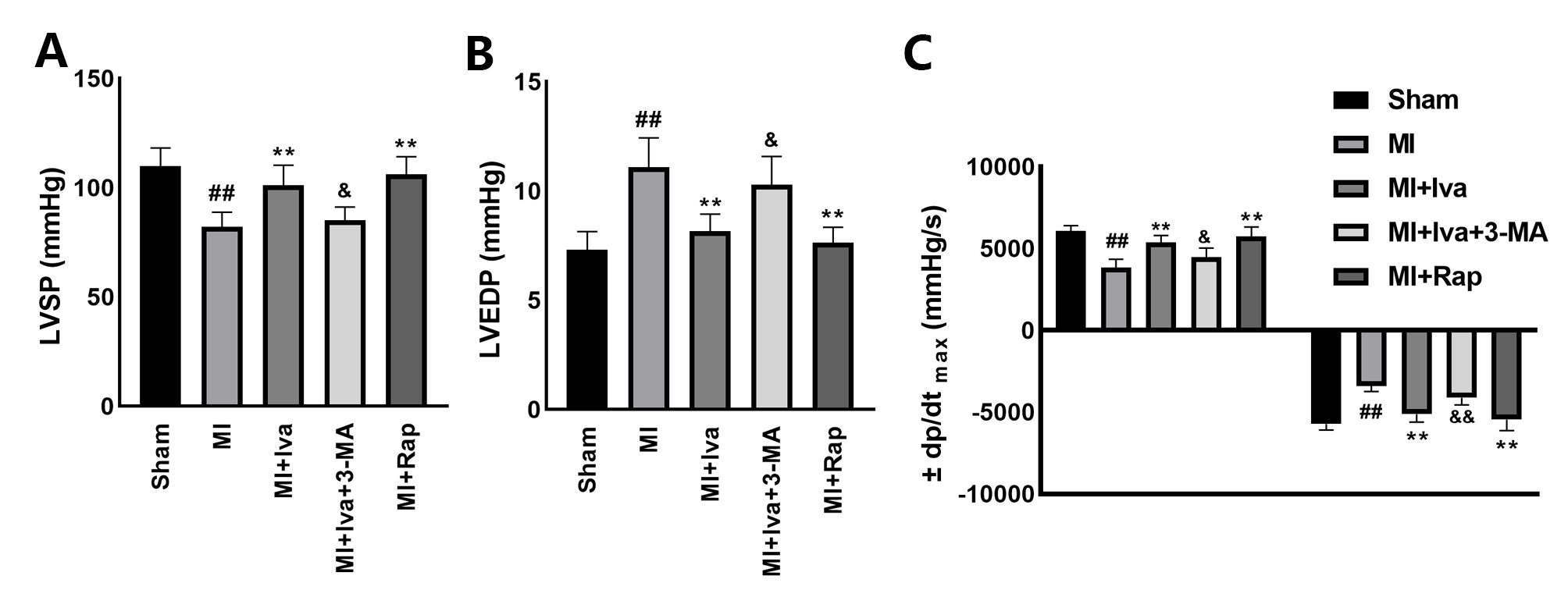

Supplement: Supplemental Material [file KBIE_A_1925008_SM3779.zip › Supplementary Figure 1.tif]
